# Hirata Syndrome in a Woman With Graves’ Disease Without Exposure to Thionamides and Summary of Cases from South America

**DOI:** 10.1210/jcemcr/luaf133

**Published:** 2025-06-24

**Authors:** Naysha Yanet Chavez Rondinel, Grecia Almendra Caycho Gamarra, Jaime Eduardo Villena Chávez

**Affiliations:** Facultad de Medicina Alberto Hurtado, Universidad Peruana Cayetano Heredia, 150135 Lima, Perú; Departamento de Endocrinología, Hospital Nacional Cayetano Heredia, 150135 Lima, Perú; Facultad de Medicina Alberto Hurtado, Universidad Peruana Cayetano Heredia, 150135 Lima, Perú; Departamento de Endocrinología, Hospital Nacional Cayetano Heredia, 150135 Lima, Perú; Facultad de Medicina Alberto Hurtado, Universidad Peruana Cayetano Heredia, 150135 Lima, Perú; Departamento de Endocrinología, Hospital Nacional Cayetano Heredia, 150135 Lima, Perú

**Keywords:** autoimmune hypoglycemia, insulin antibodies, Graves’ disease, Hirata syndrome, hypoglycemia

## Abstract

We report here the third case of Hirata syndrome from Peru, a 65-year-old woman who presented to the emergency room with obtunded sensorium caused by severe hypoglycemia secondary to endogenous autoimmune hyperinsulinism after 1 year of many episodes of postprandial hypoglycemia. Six months before admission, both arterial hypertension and Graves’ disease were disclosed, receiving nebivolol 5 mg day only. Eight days before admission, flavonoids were prescribed for mild venous insufficiency. Treatment of hypoglycemia included drug withdrawal, a fractionated diet with low-glycemic index carbohydrates, prednisone, and radioactive iodine for definitive treatment of Graves’ disease, with resolution of the condition 6 months later. She presented unilateral avascular necrosis of the hip as a complication of corticosteroid therapy. We did not identify any trigger factor for hypoglycemia besides Graves’ disease as she did not receive any drug at hypoglycemia onset. We recommend checking for anti-insulin antibodies in cases of endogenous hyperinsulinism associated with an autoimmune disease, despite the absence of any drug treatment.

## Introduction

Hirata syndrome, or autoimmune hypoglycemia, is a rare cause of hyperinsulinemic hypoglycemia. It is generally characterized by reactive and persistent hypoglycemia in patients who have never received exogenous insulin and without pathological abnormalities in the pancreatic islets [1]. It is produced by anti-insulin antibodies that initially bind to insulin and subsequently bind off, causing hypoglycemia [2].

The estimated prevalence reported in the medical literature in the Japanese population is 0.017 per 100 000 inhabitants [3]. These cases are classically reported in the Asian population and are rare in Western countries. The present case is the third one reported in Peru.

## Case Presentation

A 67-year-old mestizo woman from Lima, Perú, with a history of high blood pressure and venous insufficiency of the lower extremities without relevant family history, reported a 12-month history of drowsiness and intermittent postprandial sweating with spontaneous remission, associated with fatigue, liquid stools, and weight loss. The patient went to the emergency room with diaphoresis 1 hour before her admission, associated with facial redness and incoherent speech, with progression to loss of consciousness and relaxation of sphincters. On admission, capillary glucose was 39 mg/dL (2.16 mmol/L), corroborated by serum glucose 36 mg/dL (2.00 mmol/L), and she recovered after a glucose bolus and infusion, fulfilling the Whipple triad.

Six months before her admission to the hospital, she was diagnosed with thyrotoxicosis, without any pharmacological treatment, and arterial hypertension treated with nebivolol 5 mg/day. Eight days before her admission, she received a prescription of diosmin 450 mg and hesperidin 50 mg/day due to venous insufficiency in the lower limbs.

## Diagnostic Assessment

On clinical examination, her vital functions were blood pressure 110/60 mmHg, heart rate 73 beats per minute, respiratory rate 20 breaths per minute, temperature 36° Celsius, and body mass index 24.9 kg/m^2^.

The patient was sleepy with 13 points on the Glasgow scale. She had warm, thin skin without hyperpigmentation, and the thyroid felt firm, diffusely enlarged, and painless. Neither ophthalmopathy nor pretibial myxedema or acropachy was present. Slight distal tremor and brisk tendinous reflexes were present. The findings of the rest of the physical examination were normal.

During hospitalization, episodes of hypoglycemia recurred intermittently, predominating in the early morning, while fasting, and some postprandially. A prolonged fasting test elicited an episode of hypoglycemia in the early morning within the first 24 hours, with endogenous hyperinsulinism [insulin >1000.0 µIU/mL (>6945.0 pmol/L)] and molar ratio of insulin to C-peptide greater than 1. A high titer of insulin antibody was disclosed (Table 1); magnetic resonance imaging of the pancreas showed no evidence of focal lesions. Due to these findings, the diagnosis of Hirata syndrome was established.

**Table 1.** Laboratory tests

Test	Results	Normal range
Hematology and biochemistry		
Hemoglobin	13.1 g/dL (131 g/L)	12.1-15.1 g/dL (121-151 g/L)
White blood cell count	8.16 ×10^9^/L (8.16 ×10^9^/L)	4.5-11.0 ×10^9^/L (4.5-11.0 ×10^9^/L)
Glucose	36 mg/dL (2.00 mmol/L)	70-99 mg/dL (3.89-5.49 mmol/L)
Creatinine	0.46 mg/dL (40.66 μmol/L)	0.7-1.3 mg/dL (61.88-114.92 μmol/L)
Aspartate aminotransferase	40 U/L (0.67 μkat/L)	20-48 U/L (0.33-0.80 μkat/L)
Alanine aminotransferase	32 U/L (0.53 μkat/L)	10-40 U/L (0.17-0.67 μkat/L)
Hormones and endocrine autoantibodies		
TSH	<0.005 μIU/mL (<0.005 mIU/L)	0.27-4.2 μIU/mL (0.27-4.2 mIU/L)
Free T4	42.61 pmol/L (42.61 pmol/L)	12-22 pmol/L (12-22 pmol/L)
Cortisol	15.7 ug/dL (433.13 nmol/L)	5-25 ug/dL (137.94-689.70 nmol/L)
Antithyroid peroxidase	114.1 IU/mL (114.1 kIU/L)	0-34 IU/mL (0-34 kIU/L)
Antithyroglobulin	188.9 IU/mL (188.9 kIU/L)	0-115 IU/mL (0-115 kIU/L)
Anti-TSH receptor	2.94 IU/L (2.94 IU/L)	1.0-1.75 IU/L (1.0-1.75 IU/L)
Prolonged fasting test		
Nadir glucose	40 mg/dL (2.22 mmol/L)	
Insulin	>1000.0 µIU/mL	2.5-25.0 µIU/mL
	(>6945.0 pmol/L)	(17.36-173.63 pmol/L)
Proinsulin	>20.0 mIU/mL	0.2-1.5 mIU/mL
C-peptide	15.73 ng/mL (5.21 nmol/L)	1.1-4.4 ng/mL (0.36-1.46 nmol/L)
Ratio insulin/peptide C	>1.33 nmol/nmol	<1 nmol/nmol
Anti-insulin antibodies	>90% percentage of fixation	<8% percentage of fixation

Values in parentheses are International System of Units.

Graves’ disease was confirmed by a suppressed TSH, an elevated free T4, and a high titer level of anti-TSH receptor, antithyroid peroxidase, and antithyroglobulin antibodies (Table 1). Additionally, an ultrasonography with color flow Doppler showed a diffuse goiter with increased vascular flow. Treatment with thiamazole 10 mg/day orally was started for a few days due to the risk of exacerbation until the acknowledgement of the presence of anti-insulin antibodies.

## Treatment

For Hirata disease, the patient received glucose solutions intravenously along with a fractional diet with low glycemic index carbohydrates. After a week of such measures without improvement of the hypoglycemia, oral prednisone was started at 0.5 mg/kg per day (30 mg), with resolution of hypoglycemia and tapering off glucose infusions.

For Graves’ disease, thiamazole was discontinued to avoid exacerbation of Hirata disease, and prednisone 30 mg/day was administered for both hyperthyroidism and hypoglycemia control; 15 mCi (555 Mbq) of radioactive iodine was administered as a definitive treatment 2 months after hospital discharge.

## Outcome and Follow-up

In the outpatient setting, blood glucose improved without new episodes of hypoglycemia. After the second month of treatment, a progressive taper of the corticosteroid dose was started until its discontinuation in the sixth month. During this follow-up period, insulin and anti-insulin antibodies declined, as shown in Table 2. In the fourth month after hospital discharge, an unilateral avascular necrosis of the hip as a complication of corticosteroid therapy occurred, which was treated surgically with favorable evolution.

**Table 2.** Fasting insulin levels and anti-insulin antibodies during treatment

Month of treatment	Fasting insulin	Anti-insulin antibodies (%)
Month 0	>1000.0 µIU/mL	>90.0% percentage of fixation
	(>6945.0 pmol/L)	
Month 3	142.0 μIU/mL (986.19 pmol/L)	
Month 4	41.9 μIU/mL (291.00 pmol/L)	59.7% percentage of fixation

Values in parentheses are International System of Units.

Hypothyroidism was evident after the seventh month of radioactive iodine treatment, and the patient was started on hormone replacement with levothyroxine.

## Discussion

Hirata et al reported the first case of autoimmune hypoglycemia in 1970. The highest number of cases of autoimmune hypoglycemia were reported in Japan, China, and other Eastern countries, probably explained by the high frequency of the DRB1*04:06 allele in these populations; however, this is not exclusive to this region, since there are reports of the presence of this allele in different populations.

The incidence is rare in European and American countries [4]; in South America, there are 14 cases described so far: 6 in Brazil, 2 in Chile, 2 in Argentina, 1 in Paraguay,1 in Colombia, and 2 in Peru [5-17]; our case is the third one reported (Table 3).

**Table 3.** Reported cases of Hirata syndrome in South America

Case	Country	Sex/age	Hypoglycemia profile	Possible triggers	Comorbidities	Treatment	References
1	Brazil	F/63	Reactive	Captopril	Arterial hypertension, metabolic syndrome	Drug withdrawal	Reis et al (5)
2	Brazil	M/55	Reactive	Unknown	None	Fractional diet	Paiva et al (6)
3	Brazil	M/56	Without profile	Antibiotics probably	Sepsis	Corticosteroids, verapamil previous to subtotal pancreatectomy and hypercaloric diet	Moreira et al (7)
4	Brazil	M/6	Without profile	Unknown	Healthy	Fractional diet diazoxide, octreotide glucagon, hydrochlorothiazide	Dos Santos et al (8)
5	Brazil	F/7	Without profile	Unknown	Pneumonia	Corticosteroids for bronchospasm and a severe urticarial allergic reaction	Alves et al (9)
6	Brazil	F/59	Without profile	Monoclonal antibodies to bind to insulin	Multiple myeloma	Chemotherapy	Souto et al (10)
7	Chile	M/36	Fasting	Unknown	Healthy	Fractional diet	Lanas et al (11)
8	Chile	F/65	Reactive	Unknown	Arterial hypertension, depression	Fractional diet acarbose, corticosteroids	Lanas et al (11)
9	Argentina	F/66	Reactive	α-lipoic acid	None	Drug withdrawal, fractional diet	Izzo et al (12)
10	Argentina	F/28	Without profile	Thiamazole	Graves’ disease	Drug withdrawal	Fux Otta et al (13)
11	Peru	F/39	Without profile	Thiamazole	Graves’ disease	Drug withdrawal, fractional diet, corticosteroids	Otoya et al (14)
12	Peru	M/76	Fasting	Metformin, irbesartan, clopidogrel, paracetamol, atorvastatin, tramadol, folic acid, thiamine, omeprazole, Pfizer-BioNTech vaccine against SARS-CoV-2	Graves’ disease, arterial hypertension, type 2 diabetes, stroke sequel, rheumatoid arthritis	Corticosteroids	Teruya et al (15)
13	Paraguay	F/32	Without profile	Thiamazole	Graves’ disease, asthma	Drug withdrawal, fractional diet, corticosteroids	Galeano et al (16)
14	Colombia	M/34	Without profile	Thiamazole	Graves’ disease	Decrease in dose of thiamazole and corticosteroids (for thyroid eye disease)	Torres et al (17)

Abbreviations: F, Female; M, Male.

Regarding the present case report, in those reported previously from South America, the triggering factor was not identified in 5/14. In all 4 cases with concomitant Graves’ disease, it was associated with previous exposure to thiamazole, and in the same way as the present case, in 7/14 cases corticosteroids were also required for hypoglycemia control. Lastly, the resolution period of hypoglycemia was reported only in 6/14 and ranged between 4 and 7 months in 4 cases and 7 days to 5 years in 2 patients. This pathology affects women and men equally and is more common in patients over 40 years, with the highest peak between 60 and 69 years of age [15].

This syndrome is due to the formation of IgG autoantibodies against native insulin and proinsulin, forming macromolecules [2, 18]. The mechanism of hypoglycemia includes anti-insulin antibodies that bind to endogenous insulin and form an insulin-Ab complex; hence insulin cannot reach its receptor in the liver and other peripheral tissues, causing transient hyperglycemia, which stimulates the pancreas to release more insulin, turning this complex unstable as the antibodies lose affinity to insulin and bind off, releasing insulin molecules leading mainly but not exclusively to postprandial hypoglycemia [18].

In 1983, a case report described that the use of medications with sulfhydryl groups may trigger this syndrome [19]; since then, multiple drugs associated with this disorder have been identified, including α-lipoic acid; antithyroid drugs, such as thiamazole and carbimazole; as well as some antihypertensives (captopril, hydralazine, diltiazem, procainamide) and antibiotics (G penicillin, imipenem, isoniazid). These drugs may have a reducing capacity, which binds and breaks the sulfhydryl bonds of the A and B insulin chains, generating a molecular structural change that makes it more immunogenic [1, 19, 20].

In up to 80% of cases, an association exists with autoimmune diseases such as systemic lupus erythematosus, rheumatoid arthritis, polymyositis, and Graves’ disease; the latter is the second most common association and Hashimoto thyroiditis the third [20]. In this context, it has been identified that autoimmune polyglandular syndrome type 3A is characterized by the presence of Graves’ disease or Hashimoto thyroiditis coexisting with any of the following: type 1 diabetes mellitus, Hirata disease, premature ovarian failure, chronic hypoparathyroidism, autoimmune hypothalamitis, lymphocytic adeno-hypophysitis, and lymphocytic neuro-hypophysitis [21, 22].

The patient in the present case had symptoms of hypoglycemia 6 months before those of Graves’ disease. Initially, she did not receive treatment for hyperthyroidism until she was admitted to the hospital, when thiamazole was started and promptly discontinued when the diagnosis of autoimmune hypoglycemia was confirmed to avoid exacerbation of her clinical condition. Trying to identify the triggering factor was impossible since the patient denied any history of infections or the use of drugs reported in the literature as a triggering factor. There did not seem to be a typical triggering factor that has been reported in the literature.

The symptoms of hypoglycemia are usually mild, but severe manifestations such as seizures and coma have been described. Several case series reported clinical manifestations lasting approximately 3 and 6 months; few cases exceeded 12 months of illness [23, 24]. The patient in the present case had 1 year of symptoms of hypoglycemia before she sought medical aid.

Hypoglycemia is characteristically postprandial, as the pathophysiology of this condition shows, but it can also occur spontaneously on fasting [25, 26], as in the present case, where, before admission, the patient had episodes of mild postprandial hypoglycemia; while in the hospital setting, some were fasting too.

In Hirata syndrome, a usual laboratory finding is a fasting insulin level greater than >1000.0 µIU/mL (>6945.0 pmol/L) and a molar ratio of insulin to C-peptide greater than 1 [1, 18, 27]. The diagnosis is confirmed by detecting high levels of anti-insulin antibodies without any alteration of the pancreas, lack of previous exposure to exogenous insulin, and absence of neoplasia [28]. The patient met all these conditions.

There is no consensus on the treatment of this condition. Several alternatives are proposed, from suspending the triggering drug to establishing a fractionated diet with low glycemic index carbohydrates, corticosteroid therapy, plasmapheresis, immunosuppressants, and pancreatic surgery [16, 27-30]. In the literature, discontinuation of the triggering drug and a modification in diet are first-line treatments, with resolution of the condition in several reported cases.

Corticosteroid therapy is an alternative when hypoglycemia does not resolve with first-line treatment. Its use is based on the hyperglycemic effect due to insulin resistance and the suppression of insulin autoimmunity, and in this particular patient with Graves’ disease, it helps suppress thyroid autoimmunity and decreases the peripheral conversion of T4 to T3 while awaiting definitive treatment with radioiodine [28]. Prednisone and methylprednisolone are the most commonly used corticosteroids. The adverse events of these drugs, like the avascular necrosis of the femoral head this patient experienced, should be realized. Resolution of the condition usually occurs 3 to 6 months after starting steroids [23].

In conclusion, this patient had autoimmune hyperinsulinemic hypoglycemia manifested fasting and postprandially with concomitant Graves’ disease that fits with autoimmune polyglandular syndrome type 3A, requiring a fractional diet and glucocorticoids for resolution of the hypoglycemia and radioiodine for Graves’ disease. So far, 15 similar cases have been reported from South America, including the present one.

## Learning Points

- Hirata syndrome is the most likely diagnosis for hypoglycemia with very high levels of insulin and elevated titers of insulin autoantibodies.
- The relationship between autoimmune diseases and hyperinsulinemic hypoglycemia syndrome is well recognized. Thus, testing for insulin autoantibodies is indicated, even if a triggering factor is not identified.
- The hypoglycemia is frequently postprandial but may be fasting as well.
- Withdrawal of the triggering drug and dietary changes are first-line measures. If the condition is not resolved, alternatives include corticosteroids, plasmapheresis, immunosuppressants, and pancreatic surgery.

## Contributors

All authors made individual contributions to authorship. N.C., G.C., and J.V. were involved in the diagnosis and management of this patient and in redacting the manuscript; N.C. was responsible for manuscript submission. All authors reviewed and approved the final draft.

## Data Availability

Original data generated and analyzed for this case report are included in this published article.
